# Resident-centred care and architecture of two different types of caring residences: a comparative study

**DOI:** 10.1080/17482631.2018.1472499

**Published:** 2018-06-05

**Authors:** Catharina Nord

**Affiliations:** Department of Spatial Planning, Blekinge Institute of Technology, Karlskrona, Sweden

**Keywords:** Patient-centred care, architectural space, assemblage, assisted living, extra-care housing

## Abstract

The relationship between architectural space and resident-centred care is poorly understood, even though architectural space is indicated as an important factor in the quality of care. This paper aims to address this gap in existing research by putting resident-centred care in the theoretical context of relationality and emergence in which space is a co-producing component. This qualitative case study includes two housing alternatives, which are compared: one assisted living and one extra-care housing residence, which differ in their legal status and architecturally. Similar fieldwork was carried out in the two residences. Individual interviews with staff and residents, as well as observations—direct and shadowing—were the main data collection methods. The concept of *assemblage* was used for the analysis of how resident-centred care and architectural space co-evolved. The findings show that resident-centred care appears in similar but also diverse and sometimes contradictory ways in different spaces in the two housing alternatives, suggesting that resident-centred care is situated, volatile and emergent. Although architecture has strong agency, space and care need to be considered together—a *caring architecture*—in order to understand the nuances and rich conceptual palette of resident-centred care.

## Introduction

Resident-centred care(care that is centred on the resident, patient or the person is conceptualized differently by different authors and in different disciplines; however, they share similar traits and ideology. I have chosen the term ‘resident-centredʼ for this article because the focus is on two residences for older residents in need of care) is a hallmark of care practices (Li & Porock, 2014; Yee, Capitman, Leutz, & Sceigaj, 1999). There is no common definition of this care ideology either in practice or theory (Li & Porock, 2014), but certain shared elements can be identified. Resident-centred care is holistic and aims to satisfy the older individual’s needs, wishes and choices, provide individualized care, and strengthen his or her autonomy and self-determination (Brownie & Nancarrow, 2013; Li & Porock, 2014; McCormack et al., 2010; Morgan & Yoder, 2012; Yee et al., 1999). Resident-centred care concerns not just a relationship between a nurse or caregiver and an older person, but needs to be considered in a wider context of relations including, for instance, family, community, caring culture and, of particular relevance to this paper, the built or physical environment (McCormack & McCance, 2006; Nolan, Davies, Brown, Keady, & Nolan, 2004). This paper aims to contribute to the existing research by putting resident-centred care in a theoretical context of relationality and emergence (DeLanda, 2016). A point of departure is that care is a practice and an approach enmeshed in spatial, material and architectural circumstances (Foley, 2011; Mol, Moser, & Pols, 2010; Nord & Högström, 2017). Thus, by adopting a theory of emergence, resident-centred care is not something that *is*, but rather something that *becomes*, dependent on circumstances and co-constituting components. From this theoretical perspective, it is expected that resident-centred care may appear in various shapes and modes in different spatial circumstances.

Although authors have highlighted the importance of the physical environment for resident-centred care (Li & Porock, 2014; McCormack & McCance, 2010), the architectural environment is poorly understood in relation to this type of care or its equivalents. Few studies have focused upon this topic, although one paper focusing explicitly on the environment studies patient-centred care and hospital architecture (Bromley, 2012). Two studies on residences for the elderly have highlighted architectural qualities as factors that are expected to contribute to resident-centred care (Kane, Lum, Cutler, Degenholtz, & Yu, 2007; Molony, Evans, Sangchoon, Rabig, & Straka, 2011). However, the care model is not described in either study, even if it is referred to as resident-centred care. The two studies examined residents’ performance and perception as well as outcomes of care in small-house nursing homes compared to conventional nursing homes. In both studies, residents had moved from conventional nursing homes to small-house nursing homes. The architectural design is described in both studies: the small-house nursing homes presented a small-scale environment, private flats with bathrooms, and facilities for the community of residents. The conventional nursing homes offered double or triple resident rooms. Molony and colleagues (2011) examined *at-homeness*. The small-house nursing homes came out in a better light in most aspects compared to the conventional nursing homes. The research revealed better performance in activities of daily living (ADLs) as well as improvement in health. The resident preferences for the physical environment in the small-house nursing homes were unanimously positive. In particular, the single rooms were highly appreciated compared to the shared rooms in the conventional nursing homes. The higher degree of choice and the closer relationships with the staff in the small-house nursing homes were also appreciated. However, there were aspects which did not differ in the compared facilities, such as the attentiveness of staff, which was appreciated in both categories of nursing home. Autonomy was strongly linked to at-homeness in all facilities, although not directly associated with residential satisfaction. Kane and colleagues (2007) measured aspects such as residents’ self-reported quality of life, their self-care abilities and functional performance, which in all measurements were better than or the same as in two compared conventional nursing homes. Quality of life included aspects such as privacy, dignity, autonomy and individuality, which are often associated with resident-centred care. The study also found a higher level of quality of care. However, these were mostly assessed based on medical outcomes. The small-house nursing homes were assessed as a better environment to live in than compared facilities, though to what extent this refers to the architectural environment is not entirely clear.

These studies take as an implicit point of departure the fact that architecture has an impact on resident-centred care. However, they do not explicitly discuss the architectural conditions or spatial impact, and this is the gap in existing research that this paper aims to address.

### Assisted living and extra-care housing in Sweden

**Figure 1.**
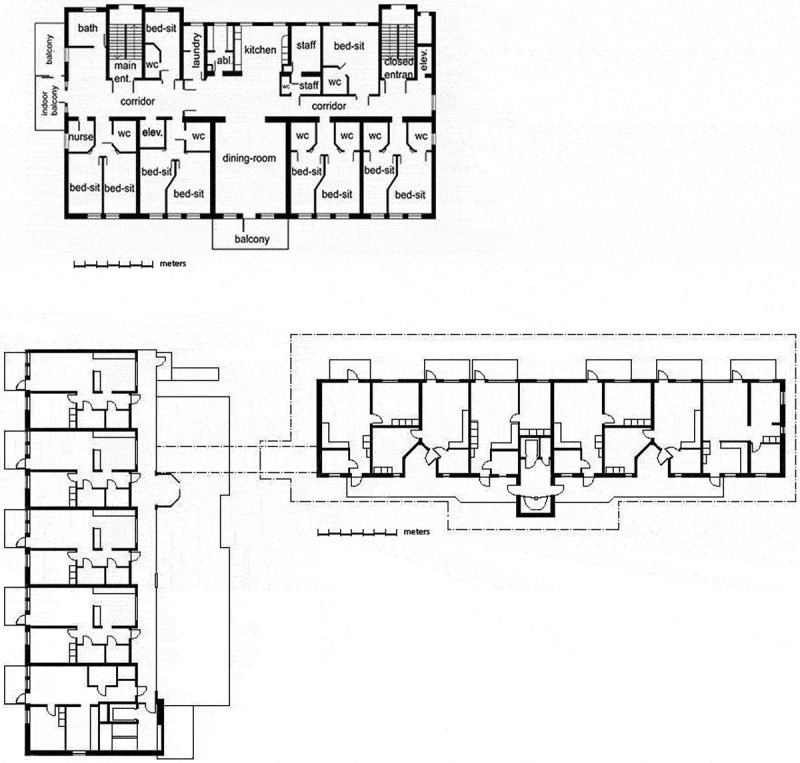
Plan of assisted living facility and extra-care housing (below). In comparable scales.

This study analyses resident-centred care in two different architectural layouts of housing for elderly people with caring needs in Sweden: assisted living and extra-care housing. Assisted living is a right-based housing option for older people under the Social Services Act, provided by the municipality in the event that the person is assessed as needing 24-hour care (SFS, 2001, p. 453). There are considerable spatial similarities between all newly built assisted living facilities. As a rule, they consist of a corridor with small bed-sitting rooms and common spaces for dining and socializing (Nord, 2013; Regnier, 2002). Each elderly individual rents a bed-sitting room of about 30 m^2^, including a bathroom, hallway and a kitchenette. This design is a materialization of a collective-care model where the residents are supposed to take part in common meals and activities (Nord, 2017). The facilities are staffed around the clock. The assisted living facility in this study is a reconstructed nursing home adapted to the building regulations, and, as such, a representative of this building type, presenting a similar design (Figure 1).

The second case in this paper is an extra-care housing residence (Figure 1). It differs from the assisted living facility in important respects, such as architecturally, by offering full one-bedroom flats to the residents. Nor is it subject to the same legal restrictions of access as the assisted living facility. In other respects, it is similar: it has common rooms, a dining facility and a living room. It is also a housing alternative with permanent staff for people with greater care needs, similar to those in the assisted living facility. These design differences are the point of departure for the analysis of resident-centred care, which is expected to bring out nuances of this type of care. It is anticipated that it will give insight into the ways architectural spaces give shape to resident-centred care in two forms of housing which are adapted to older peopleʼs needs.

### Assemblage theory

The main analytic question is *how does resident-centred care emerge in architectural space in these two different housing alternatives*? The theoretical point of departure for this study is the concept of *assemblage*, which refers to a constellation of heterogeneous components that interact by forming associations and relations (DeLanda, 2006, 2016; Deleuze & Guattari, 2004). Components included are of different orders and types—human or non-human—structured by self-organizing processes; a whole that has emergent properties. Thus, an assemblage is constantly moving and changing. *Emergence* and *becoming* are two central concepts in this theory, indicating how different components are shaped in processes of interaction (DeLanda, 2006, 2016). The concept of assemblage has been applied to architecture in the sense that architecture is an assemblage formed by its included material, human and spatial components (Dovey, 2013; Jacobs, 2006). Assemblage theory endows dead material with agency. Thus, the materiality of architectural space is an important agent in these assemblages (cf. DeLanda, 2016; Duff, 2014). Architecture does things to people (Gieryn, 2002). However, as Brott alerts us: “This does not mean that cities, buildings or interiors become persons, but … [l]ived experience is altered as a result” (Brott, 2013, p. 3). When different architecture impacts on other components such as residents and staff, it is expected that the lived experience of resident-centred care will emerge in different shapes and modes. For instance, in this case, architectural agency distributes staff and residents in different spaces, which may affect their relations. These are processes that occur the other way around as well. Even though architectural space is often well defined by walls, ceilings and other building components, it nevertheless gets its shape and meaning from its interaction with people and non-human components. A room is formed by the practices and habits of the person who is living there. Thus, architectural space does not provide a spatial container in which care practices are carried out, but becomes a *caring architecture* by co-producing quality, personal relations and negotiations of routines in the practice of care situated in space (Nord & Högström, 2017). In this study, the differences and similarities between the two residences compared are expected to form different assemblages in which resident-centred care will be enmeshed and moulded.

## The methodology of the study

This study is a case study that applies qualitative methodologies to grasp the messiness and heterogeneity in data collection and analysis (Lather & St. Pierre, 2013; Law, 2004). Individual interviews and observations were the primary methods of data collection. They were adapted to assemblage theory by identifying relations between care work and architectural space using *transcendental empiricism* (Duff, 2014). This methodology aims to empirically ‘investigate a given assemblage to demonstrate how it is composed and the specific causal mechanisms by which social and/or structural processes enter into it’ (Duff, 2014, p. 55). Thus, by considering the two residences as assemblages, it was possible to identify their various components, and associations between these components, as well as processes going on in which resident-centred care appeared.

### Fieldwork organization

Similar fieldwork was carried out in the two different housing alternatives, an assisted living residence (18 months) and an extra-care housing residence (10 months), in order to perform a comparison. The first period of fieldwork, in the assisted living residence, was more time-consuming, due to its more explorative nature, than the second, in the extra-care housing residence, where it was possible to focus more intently on obtaining data. The field study started out in both residences with a period of observation of about one to one-and-a-half months. This period was intended to provide an understanding of everyday life in the two residences and generate topics to be raised later in interviews. After interviewing started, observation continued, alternating with the interviews. This way of organizing the fieldwork made it possible to compare and co-analyse data from the two methods throughout the work in order to develop new issues and to check whether people’s self-reporting of their activities was accurate. Space is everywhere and is a self-evident framing to everyday life, so it tends to go unnoticed. People may have difficulties in reporting what they do in space because they are enmeshed and embodied in spatially situated situations (O’Toole & Were, 2008). Fieldwork was divided into sessions; that is, visits several times a week when the fieldwork was most intense. Each fieldwork session lasted four to six hours during daytime. In total, the fieldwork amounted to about 145 hours in the assisted living residence and 100 in the extra-care housing residence. The author participated to some degree in the care work, but only in tasks where there was no risk of affecting residents with any possible mistakes (Adler & Adler, 1994).

### Data collection methods

Observation occurred in two ways: as direct observation of everyday life in the residences (O’Toole & Were, 2008), and as shadowing (Czarniawska, 2007). The selection of resident participants for observations was discussed with the staff in order to exclude people who were, for example, too weak, or for other reasons. One person was recommended for exclusion because of his aggressive moods. (The person in question was later included in interviews because the author connected well with him.) The ambition was to include as many residents as possible, men and women of different ages and with different conditions, in order to cover as many types of caring situations as possible. The residents were approached by the author in the company of a staff member and asked in person whether they wanted to participate. One declined and was thus not approached anymore. The direct observation comprised various situations. The space where these situations took place and the people and things involved were observed and noted. Shadowing also involved the recording of situations in space but comprised the accompanying of care workers while they worked, observing the work that they carried out and the different types of objects they used in different spaces. Thus, in the latter, the focus was on work situations while the former could include any situation, such as interactions between residents. Both types of observations were documented with detailed field notes, and the physical environment was documented with photography and drawings. I also interacted frequently with the staff as well as the residents, both individually or in groups, and had many informal conversations with them. Furthermore, individual interviews were carried out with the majority of the staff members; 15 in the assisted living facility, and 16 in the extra-care housing residence, including the unit managers. Eight residents were interviewed in each residence. Both groups of interviewees participated voluntarily, on condition that participation was anonymous. Only residents with full cognitive capacity were asked to participate, in order to ensure that they fully understood the implications of participation and the study aims. People who were very fragile were also excluded. The ambition was to include as many staff as possible, so all staff members who wanted to be interviewed were included. All agreed but two were later excluded because of long-term sick-leaves. This gave naturally a mix of men or women staff members, although the latter was in the majority. Interviews lasted around one hour for staff, and half an hour to one hour for residents. Staff interviews were carried out in a staff room where no one else was present apart from the interviewer and the interviewee, and the residents were interviewed in their flats. All interviews were carried out in Swedish during the daytime, were recorded with the consent of the interviewee (no one declined) and then transcribed verbatim by a professional transcriber.

### Analysis

Using transcendental empiricism, data from the various methods were drawn together into wholes, forming rich and multifaceted assemblages of situations in space in which people and materialities were immersed. By applying transcendental empiricism it is possible to investigate how concepts vary according to the context in which they are articulated (Duff, 2014). The analysis in this study builds on DeLanda’s notion that relations of externality make it possible to move the concept of resident-centred care from one assemblage to another (DeLanda, 2016) and, thus, to explore how it changes and varies. Deleuze & Guattari (1994, pp. 15–16) assert that

[t]here is no simple concept. Every concept has its components and is defined by them…there is no concept with only one component … Every concept has an irregular contour defined by the sum of its components, which is why, from Plato to Bergson, we find the idea of the concept being a matter of articulation, of cutting and cross-cutting.

These ontological assumptions were a guiding principle for the analysis. The two cases were analysed in two steps. First, a separate analysis of the two housing alternatives was completed that aimed at articulating co-constituting central concepts of resident-centred care which were developed from the existing literature presented above: (1) resident needs, wishes and choices, (2) individualized care, and (3) residents’ autonomy. This analysis aimed to reveal the various assemblage components related to these concepts and their associations and relations, in order to give shape to the contours of the concept of resident-centred care in the two situations. In the second step, the results of the analysis of one of the two housing alternatives were compared, in which interlinks, similarities and differences were defined by conceptual variabilities with a special focus on architectural space.

### Ethical considerations

This study received ethical approval from the Ethical Board in Linköping, Sweden (no. 2015/335–31). An ethical approval is, however, not sufficient; carrying out research with weak older people demands an ethical attitude throughout the study (Vetenskapsrådet, 2002). Even though the participants had agreed initially to participate anonymously and voluntarily, according to established ethical procedures, I asked repeatedly at every visit to individual flats whether the resident approved of my presence (Loue, 2002). In particular, the shadowing technique, in which the researcher may closely approach a resident’s sphere, demands repeated consent (Czarniawska, 2007). Changes in the residents’ attitudes were also noted during shadowing; on one occasion it was interrupted and the author left the flat when the resident explicitly disapproved of her being there.

## Results

**Figure 2.**
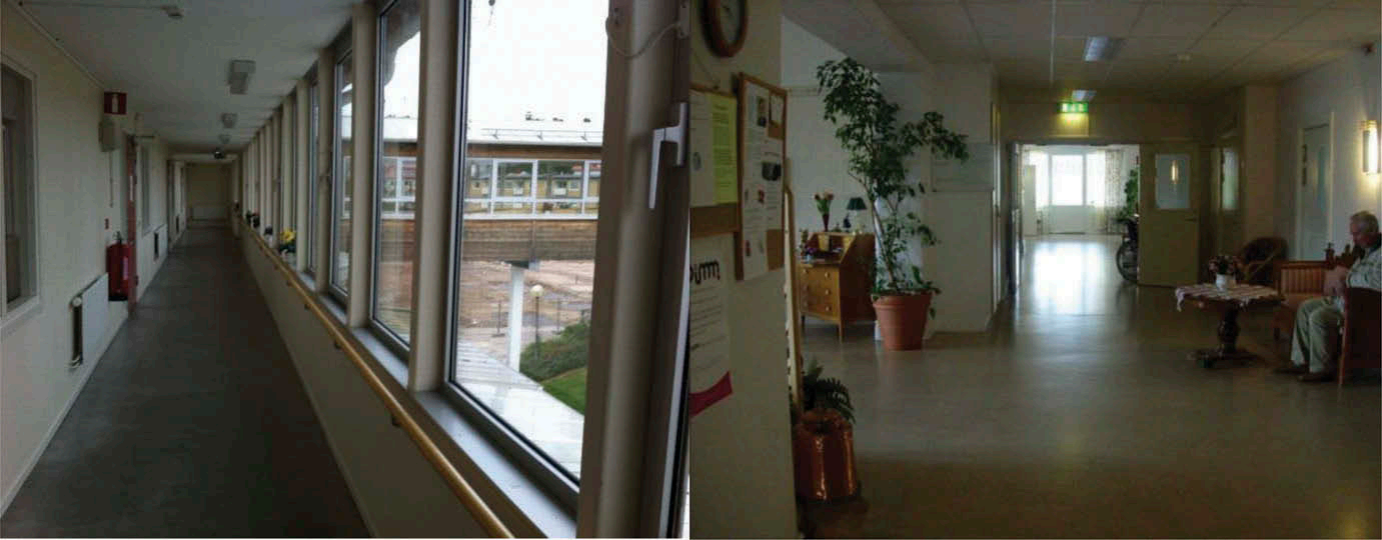
Corridor in the extra-care housing and the assisted living facility (to the right).

The two housing alternatives in the study can be understood as two architectural assemblages formed by spatial and material components and their associations, giving rise to the emergence of distinctly different environments. While the assisted living facility was a restricted, dense assemblage with weak or non-existent boundaries between private and public spaces, the extra-care housing residence was spread out, with strong demarcations between private and public space (Figure 2).

The high density of the assisted living residence was produced by the fact that there was a view throughout the whole unit, which was a restricted area on one floor with short distances between rooms and people, and often open doors to the residents’ rooms. There were 13 residents, with no one below 80 years old. The oldest turned 98 during the study. The staff to resident ratio was 1:1, including the night staff, and about 3–6 staff members worked during each shift. The fact that staff often worked in twos when assisting one resident added to the density. They planned their tasks together and did not follow any written caring protocol but provided the care they considered essential to satisfy the resident’s need. The resident bed-sitting rooms were small, and there was a common room, furnished for dining and watching TV. This integration of spaces and people contributed to blurred borders between private and public areas.

The extra-care housing residence was a three-storey building with long distances between the doors to the flats, which were closed as a rule. This dissociation of spaces made it impossible to have a view of the whole housing block at any point, and the environment had clearer demarcations between private and public areas. There were 24 residents, of whom the youngest was 62 and the oldest 99. The number of staff was similar to that of the assisted living residence. The staff to resident ratio was about 1:2, including night staff. Every staff member received each day an individual list of tasks and scheduled visits to the residents, indicating the person visited, the care task and the allocated minutes for this care work. Staff members often worked alone and could virtually “disappear”, distributed throughout the whole building.

The following analysis aims to explore how resident-centred care appeared in these two distinctly different assemblages by investigating the three aspects of resident-centred care adopted from the literature: (1) resident needs, wishes and choices, (2) individualized care, and (3) residents’ autonomy.

### Residents’ needs, wishes and choices

The assemblages of the two housing alternatives had very different capacities to attract residents. Nevertheless, they had in common that 24-hour care was available in both and that access to care was an important factor contributing to the attractiveness of both housing alternatives. Resident needs, wishes and choices with regard to access to care came about as effects of legal and architectural components in the respective housing assemblage. One self-evident aspect of the residents’ choice of housing was whether it was possible for the individual to decide whether or not to move in. The extra-care housing was free to access based on the individual’s self-assessment of their need for care, whereas the Social Services Act associated with the assisted living made it accessible only if the individual was in need of 24-hour care, based on an assessment by a municipality officer. This difference had a great impact on the clientele in the respective assemblage. Another difference was in the design of the architectural space.

#### Extra-care housing

In addition to generally free access, a spatial analysis and direct observation showed the architectural impact on the residents’ choices. The apartments in the extra-care housing residence were flexible enough to accommodate anyone who wished to stay there. In terms of architectural agency, it is possible to say that the space invited a multitude of residents by attracting residents with a great variety of needs to the assemblage. Hence, the housing offered a high degree of resident choice. Access to 24-hour care made it possible for those with very severe disabilities to choose this housing alternative, and residents with full bodily capacity were welcome as well. Residents in the extra-care housing residence in the study thus varied greatly in health status. One man had suffered a stroke that had left him completely dependent on assistance to perform daily tasks. In contrast, two women who lived in the residence needed very little help but both had chosen to move in because they felt isolated after the loss of their spouses. One of them expressed her satisfaction with the residence in the interview:

Yes, they come and check up on me, and I am grateful for that, and then we sit down on the sofa and chat for a quarter of an hour or so. But they don’t need to help me with anything. I try to manage on my own.

The individualized care needs of all of the residents were distributed between two poles: a substantial need for help and almost no need for help at all. The most common help the residents received was with medication, meals, personal hygiene—in particular, showers—cleaning and laundry services, supervisory visits and accompanied walks outside to shops or elsewhere.

Although the need for help varied among the residents, the care offered was an important assemblage element, with great attractive force contributing to facilitating the residents’ choices to come and live in the residence. A man answered the question why he had moved to the extra-care housing: “I wanted some help … but now I have it from the girls here.” A man who was part of a couple interviewed considered the wish to access care to be an almost self-evident aspect of the move: “Of course [access to care was a reason]. Now [name of wife] gets help with showering every week.” His wife added: “And cleaning every other week.” Resident-centred care appeared to have the double capacity of being an attractor of residents from the exterior while emerging according to residents’ needs in the interior of the assemblage.

#### Assisted living

The heterogeneity was much less pronounced at the assisted living facility, due to the legal constraints, which restricted its capacity to attract a variety of residents. Similar health status and care needs of residents resulted in a more homogeneous assisted living assemblage than in the extra-care housing residence. Resident-centred care varied less, since all of the residents were very old and fragile, and the great majority required support with most personal care, such as hygiene, dressing, medication, mobility and sometimes eating.

Resident choices were an ambiguous category due to the legal embeddedness of this particular housing option and because of the residents’ health circumstances. Severe health conditions or frailty had forced them to apply for residency, and, when a sufficient level of need for care had been confirmed, they had been offered residency. The study results show that their capability to choose was severely circumscribed, and almost non-existent. One woman who stayed in a single room with her husband had vague ideas about why she had been offered a place, possibly indicating that she was not involved in the decision to move:

“I actually don’t remember why I ended up coming here; I don’t know it and it just sort of happened that I was kind of placed here when I could not manage on my own.”

It is important to point out that at the time of the interview the woman had no diagnosis of cognitive decline, and she appeared self-contained and answered the interview questions logically and thoughtfully. It seemed that she had been under such stress during her move that she reacted with confusion. Another woman remembered the process of moving, but in the interview, she expressed that she had not influenced the decision to move and that she regretted the loss of her former flat. She said:

We had a good apartment. But then we could not live there any more … We, or I, simply wasn’t allowed to live there. It did not suit me. So we had to move out of the apartment. But it worked out. It had to work out.

Comments of this type from the residents indicated more or less explicitly a lack of choice and involvement in decision-making.

**Figure 3.**
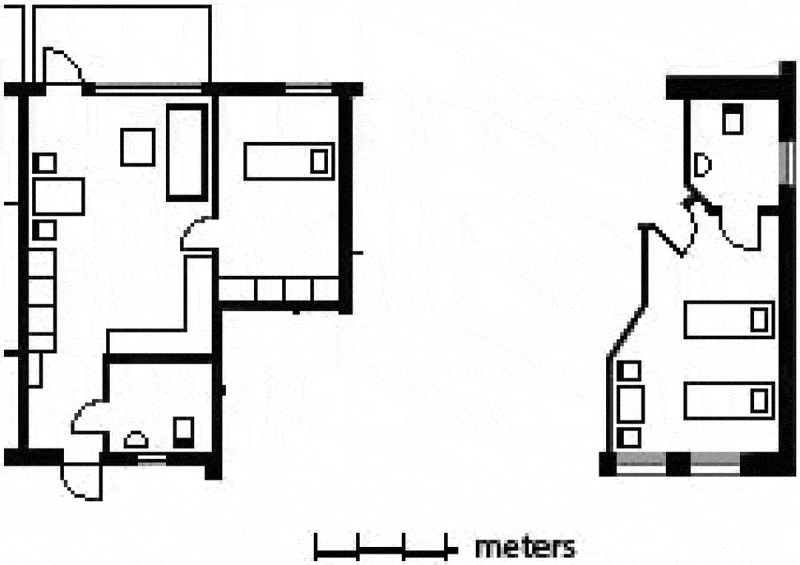
Plan of flat in the extra-care housing and the bed-sitting room in the assisted living (to the right).

The non-choice in assisted living also included the acceptance of a low-quality flat, which suggests a more or less coerced association to the assemblage. Research has also shown that residents tend to overrate the quality of the accommodation in order to justify their (non-)choice to move to assisted living (Nord 2016). This was also the case in this study. Residents said that they were pleased with the bed-sitting room of about 25 m^2^ they had been given, although the choice had explicitly been a non-choice. The wife from the interview above continued: “But seeing as I didn’t have a choice and I was forced to move, this is the best imaginable.” The bed-sitting room she and her husband shared only had space for two beds and not very much more (Figure 3). She added: “This was the only room available. We had to take it, and we did so with light hearts, so that we got somewhere to stay.” The final line of that statement indicates the precarious situation in which the couple found themselves when a move to assisted living became urgent due to increased care needs. They had to accept a very low standard of living in order to have somewhere to reside where they could access the care the woman needed. Nevertheless, the woman expressed satisfaction for this in the quote.

The other woman interviewed also expressed satisfaction with her room, but the interview revealed several reservations. When I asked which one was her favourite room, she said: “This one”, referring to her bed-sitting room. Later in the interview she elaborated further on that issue:

“Of course, my bed-sitting room is not in the best condition, but I don’t let it bother me. You have to overlook some things, you cannot start demanding that they repair and renovate. So I’m happy.”

Both of these women seemed to have accepted a low living standard in exchange for care, and they curbed their complaints by stressing how pleased they were with the situation over which they had little influence. Resident wishes and choices of housing were pushed into the background of their pressing need for care. This indicates that the move involves negotiation between the needs of disabled bodies, access to care and housing alternatives. In this case the women had chosen to associate themselves with a small bed-sitting room but where resident-centred care was available.

#### Extra-care housing

Negotiations of this kind were obvious also in the extra-care housing residence but here the capacity to choose and barter was somewhat stronger than in the assisted living facility. Similar to the results in the assisted living facility, a flat that would not be accepted under circumstances where there were no care needs may become a valid alternative when care needs increase. However, the results indicate processes of changing relations between components, such as the housing the residents had left, their increasingly recalcitrant bodies and new housing alternatives. Most had moved because their former homes had become a challenge in one way or another. A man talked about the hilly streets from the town centre to his former home, which had been increasingly difficult to manage until one day when it was not possible any longer. In one case, a woman had moved on the initiative of a municipality officer as an alternative to home-care services.

The extra-care housing offered small but full flats with kitchens or kitchenettes. One could expect more positive resident assessments of these than of the accommodation in the assisted living facility, since they were bigger and better equipped (Figure 3). However, this was not clearly the case. Most of the interviewees were somewhat reserved in their assessments of their flats. They used words such as “OK”, “decent”, “good enough”. “Well, in general there is nothing wrong with it [the flat]”, the man who was part of the couple said. When asked his opinion about the flat, another man emphasized his appreciation of the services and the kind staff, rather than the quality of the flat. His avoidance of answering this question may demonstrate the link between acceptance of lower housing quality and access to care. A woman described her flat thus: “It is peaceful and good enough, I suppose.” Most of the residents interviewed had faced the situation that only one flat was available. One male resident, who lived in one of the smaller flats with a bedroom and a combined living room and kitchen, aired severe criticism of his flat. He evaluated the components of his “flat” assemblage in material and spatial terms when he said: “This is not a flat. It would have been nice to have a kitchen. I don’t know what to call this: two rooms and a stove or something?” This man had been afflicted with a severely disabling condition before he turned 60, and thus found himself in a situation similar to the people who had had to move to an assisted living facility. He expressed the incapacitating effect disabilities have on the ability to choose and how confusing it is to cope with a situation of newly acquired disabilities:

Really, you don’t choose … It came up that “Now we should have a care conference” and I didn’t know what that meant … Perhaps I had mentioned that I might want to stay in [name of present residence] because I knew that this [housing] was here … if the option came up I could take that.

The capability of the extra-care housing alternative to accommodate a variety of care needs was an option for this man, so he was not coerced into “choosing” the assisted living alternative at the age of 58.

#### Emerging housing quality

These results suggest that perceptions of housing quality were emergent based on negotiations between access to care and housing. The residents’ chilly opinions about their comparatively spacious flats in the extra-care housing residence possibly indicate that the bed-sitting rooms offered in the assisted living facility would not have been an alternative for most of these residents at this point. The fact that many could have remained in their former homes with home-care services made them less prone to make big sacrifices regarding housing quality in order to access care. However, the relative importance of the components in the assemblage of housing and resident-centred care changes with increasing care needs. At a later point, when access to care may be a question of life or death, an individual is prepared to move to a substandard flat in order to access that care.

### Individualized care

The staff interviews in the assisted living facility revealed a trajectory of routinized care over the course of a day in which staff made an effort to respond to each elderly individual’s wishes and needs. Routine care tasks in nursing homes are mediated by personal and situational factors (Essén, 2008). Not to underestimate the importance of these adaptations of routine tasks to individuals’ needs, interviews and observation revealed that much of the individualized care work took place *at the same time or in between the routine tasks*. This care work is deeply embedded in the assemblage of human and non-human components and emerges as much less visible and more difficult to trace due to its vagueness and heterogeneity. It appeared from the contingencies of the situation and fed on resources available in the assisted living assemblage, such as architectural space. The residentsʼ private flats or bed-sitting rooms were important architectural agents contributing to these processes in both housing alternatives. Individualized care in the form of conversations between the residents and the staff appeared as a significant part of care in these rooms, and offered an opportunity to build personal relations. One staff member in the extra-care housing facility stated in the interview:

I have cleaned in John’s flat now, for half an hour or so. I think it is really nice and cosy to be able to talk to him and make some jokes. Yes, it is me and him, then and there.

Similar comments came up in the interviews with staff in the assisted living facility. Conversations seemed to grow from the contingencies at the very moment in which they took place. They could be very mundane, and with or without any particular topic, such as in this description:

“Well, you chitchat a little bit and I get to know about his children and grandchildren when they come … Then I also see to it that he can attend the activities he likes.”

It could also be an opportunity to comfort a distressed resident:

“You don’t know what to do when she gets her panic attacks at night … Well, you can sit and talk for a while and pat her and so on.”

In the assisted living facility, it was once observed through a chink in the door how a staff member was comforting a newly widowed woman in her bed, hugging her and holding her hands. Situations of this kind suggest the emergence of micro-assemblages of care of limited endurance embedded in greater assemblages of space and materiality; in this case a bed, a staff member and a mourning widow in her private room.

While get-togethers such as common meals did not generate much conversation between residents in either of the two housing alternatives, it seemed as though the peacefulness of the private flat and a meeting between only two people formed the small-scale assemblage necessary for the emergence of the residents’ ability to express themselves. When this happened in a severely disabled resident with a speech impediment, it created joy and satisfaction in the staff member who experienced it:

“Sometimes in the afternoon when you enter his room he is lying down, watching TV. Occasionally he has said that a programme was interesting and then … you are almost taken by surprise; [laughter]; then it is fun.”

The last comment, about being surprised, suggests that in between scheduled routines there is a flow of emergent events that are volatile and perhaps easy to miss. Conversations were in a constant state of becoming, depending on the staff’s abilities to seize the opportunity to enter into communication with the resident. A difference between the extra-care housing and the assisted living residences that shaped these conversations was the easy access to the residents’ rooms in the assisted living facility, in contrast with the scheduled visits in the private flats in the extra-care housing residence. The dense architectural assemblage with short distances and often open doors in the former offered emergent opportunities for peeking in while doing something else. In an interview, one staff member commented on her occasional interaction with one of the residents:

Like for example Anna; she is so anxious in the evenings, so I tell her “I’ll come back to you before I go home. And I might not come inside but I’ll open the door a little to see if you are asleep, then I’ll just leave, and if you’re awake I’ll come in and say goodbye, because I’ll be on my way home.”

These examples above show that resident-centred care may emerge as short-lived opportunities to grasp and cultivate as individualized care for a few moments. The “messiness” of encounters between staff and residents nurtures individualized small-scale care which was of great importance in the assisted living facility. The fact that the staff worked alone more in the extra-care housing residence was also a component that offered scheduled and thus recurrent opportunities for private encounters with residents. However, one staff member in the extra-care housing residence who also had experience working in assisted living thought that the frequent improvised encounters with residents in the assisted living encouraged a tighter relationship with the residents than in the extra-care housing. Although it may be a question of individual judgement in which type of residence the best relations develop, it is clear that the different physical environments and the organization of care contributed to the emergence of different kinds of staff/resident relations. In the assisted living facility, encounters between caregivers and residents were more or less unanticipated happenstances interwoven in a flow of frequent care events situated in many different spaces, while in the extra-care housing residence they were clearly chiselled-out situations more firmly emplaced in the residents’ flats, identified, appreciated and expected as such by the staff members as well as the residents.

### Residents’ autonomy

Because architectural space in the two housing alternatives contributed to two different communities in which encounters between residents and staff emerged in distinctly different ways, they offered different degrees of autonomy to the residents. In the extra-care housing residence, the residents were expected to live independently with a higher degree of autonomy, and this was encouraged by the clear boundaries between private and public spaces and the care work organization, forming an assemblage where independence could grow. As was mentioned above, there were few or no visits from the staff between the scheduled caring visits. Thus, the residents’ flats were assemblages in which solitude and privacy were nurtured. Residents, even those with severe disabilities, retained responsibility for their lives. The severely disabled man with the stroke was also expected to live independently. He was supported in this by his wife, who visited him regularly and cooked meals in his flat.

In the assisted living facility, on the other hand, support from the staff was never far away, creating an assemblage with emerging opportunities for meetings and assistance. One staff member commented in the interview on the regular presence of staff in the public areas:

“Yes, there are very often [staff] in the kitchen, actually. And we run and there are short distances, everything is close, so it is pretty certain that staff are here. Perhaps that makes residents feel safer.”

One example was a woman suffering from dementia who became very upset from time to time if she was alone in her room, and expressed her distress very loudly. When the staff took her out and she sat in her wheelchair watching them work, according to observations she seemed to become less stressed and she was able to settle down. It seemed important where in the architectural assemblage she was situated and with whom she could associate.

The corridors in the two housing alternatives were architectural spaces which gave rise to different kinds of individualized care work and meetings between residents and staff. In the extra-care housing residence, observations revealed that the corridor was mostly empty, with the exception of staff assisting residents to and from meals and activities, while in the assisted living facility, the doors to residents’ rooms were often open and a variety of care situations took place much more frequently there. Hence, the corridor had a more pronounced capacity to link the individual bed-sitting rooms to each other by the involvement of staff and residents, and contributed in these situations to the dense assisted living assemblage. Residents and staff often moved around in the corridor on a daily basis. One woman explicitly wanted to have a connection with the corridor outside her bed-sitting room. She said that she wanted to have her door open “so that I can see the hustle and bustle outside”. She may have felt more included and safe by having her door open, being more tightly associated with the greater assemblage of space, staff and other residents in the whole facility. A more dramatic event took place when a resident attempted suicide in the corridor and the staff came rushing to his succour. It is highly likely that this man took advantage of the connecting capacity of the corridor and calculated that someone would most probably see what he was doing. This would not necessarily have been a successful strategy in the extra-care housing corridor, where people passed regularly but not very often, offering much fewer emerging associations due to less supervision.

The architectural environment and the staff care work thus formed a community with tight relations in the assisted living facility, in which the residents could to some extent dispense with their autonomy and leave their safety and choices in the hands of the staff. This was highly beneficial to people with dementia, as was indicated in the situation with the yelling woman described above, while the freedom and responsibility demanded in the extra-care housing did not always work well with the needs of a person with dementia. These problems manifested in different ways when these individuals became increasingly dissociated from the assemblage due to their progressing disease, which invariably redefined the components in the assemblage and gave them new emergent and sometimes terrifying meanings. One man had created a small micro-assemblage of himself, a bed and bedding behind a closed bedroom door in the extra-care housing residence. He seemed to be horrified by the “autonomy” he was supposed to assume, and lay in his bed waiting for the staff to come. He whined from beneath his blanket: “I have been waiting for you the whole day.” A woman experienced the extra-care housing quite differently because the assemblage of the housing could not keep her contained, due to her disease. The architecture was open, with weak or non-existent borders between buildings and the surroundings. She started leaving the residence at night, since her perception of day and night was in a process of deterioration and the supervision was not sufficient for her needs. One night, a stranger who had found her in town brought her back in his car. Obviously, her safety was endangered. Both of the people afflicted with dementia moved to assisted living during the fieldwork, having become completely dissociated from the assemblage.

## Discussion

The importance of architectural space for the provision of resident-centred care has been a point of departure in existing research, although without any particular analysis or discussion (Kane et al., 2007; Molony et al., 2011). This study shows how architectural space contributes to the *emergence* of resident-centred care, and the results show that it appears in different forms in different architectural environments. Rather than ascribing stable and essential qualities to the environment, such as that this environment is good or that environment is bad, the theoretical framework of assemblage made it possible to discern care and space in becoming, and the outcome of relationships with co-constituting heterogeneous components such as laws, bodies and artefacts—a caring architecture (Nord & Högström, 2017). This resonates with theorists who contend that resident-centred care is not composed of relations between residents and staff alone, but involves material factors such as space (McCormack & McCance, 2010).

Architectural space appeared in this study as having various agentic capabilities, sometimes involving staff or other components, sometimes with a strong agency almost of its own (cf. Brott, 2013). However, *care was always a co-constituting component*. Sometimes the residents’ perception of their need of resident-centred care was a sufficient component. The latter indicates that resident-centred care is immersed in relations that do not necessarily involve staff at all. This was the case when the residents faced a number of choices over which staff had little influence, such as the individual choice to move in, or the choice of a flat or a bed-sitting room in order to get access to care. These choices arose as a significant aspect of resident-centred care, since they were an outcome of each individual’s negotiations regarding his or her individual care needs. Choices grew out of circumstances. Thus, the relationships between perceived health, material, legal, discursive and spatial components were more influential than the staff.

The contrary appeared in situations in which staff were strong co-actors together with architectural space. The study results indicate that the staff were enmeshed in spatial and material circumstances, influencing the quality and character of their provision of resident-centred care. This appeared in a number of planned and unplanned care encounters with residents in which care could develop in unpredicted ways according to contingencies then and there. Different spaces had similar or different capacities in the two residences. The residents’ flats and bed-sitting rooms were on a par in many situations, both offering material and spatial circumstances for a rich palette of care encounters, perhaps because they minimized the number of actors, to the advantage in particular of frail residents. Very small-scale events emerged as individualized incidences of high-quality resident-centred care. At a larger scale, in which the corridor of the building was a major architectural agent, the density of the assisted living proved to be more prone to connectivities and care events in becoming. In these situations of openness, in which various trajectories of care were available, lay the possibility to adapt the care to the individual’s needs, to offer individualized care (cf. Essén, 2008). In this study, autonomy appeared as a product of architectural space and the organization of care, involving relations between both flats and corridors. Autonomy and privacy were strongly linked (separate flats) and involved a lower degree of supervision in extra-care housing compared to the density of the assisted living, where residents could refrain completely from autonomy if they preferred to. This is in line with Kane and colleagues’ study (2007) in which autonomy and privacy were aspects of quality of life which was found to be higher in the small-house nursing home with private flats. This is a reasonably direct and discernible cause–effect mechanism. It is surprising, then, that the perceived autonomy the private flats offered to the residents did not appear as a factor influencing residential satisfaction in the small-house nursing homes in Molony and colleagues’ study (2011), although it affected at-homeness. The fact that perceived autonomy in conventional nursing homes in the same study was not linked to residential satisfaction is less surprising if the care needs of the residents are taken into account. This study shows that the architectural impact on autonomy was much more indirect, intangible and negotiated with care in assisted living. If residents were happy about refraining from privacy due to their care needs being satisfied by a higher degree of supervision in a dense architectural environment, and if they nevertheless perceived their autonomy as sufficient, they would most probably not see this link between autonomy and architectural space and, thus, it would not influence their residential satisfaction. This shows the elusiveness of space as taken for granted in everyday activities (O’Toole & Were, 2008). The agency of architectural space can be almost invisible.

The small-house nursing home in Kane and colleagues’ study (2007) was assessed as a better environment to live in than compared facilities. It is unclear to what extent resident-centred care contributed to this. The results in this study show that the residents’ assessments of their flats were not necessarily congruent with an objective assessment of their quality. The assisted living home environment with smaller flats came out in a better light in the eyes of the residents than was the case for the residents in the extra-care housing residence, because it seemed to satisfy their pressing needs for care. This somewhat surprising result, which contradicts the results of Kane and colleagues (2007), points to the importance of the co-constitution of care/space. Molony and colleagues’ study (2011) also showed strong resident preferences for the physical environment in the small-house nursing home. In particular, the single rooms were highly appreciated compared with the shared rooms in the conventional nursing home. In the present study it seemed that the residents had a tendency to overrate the quality of the accommodation because it gave them access to care (cf. Nord 2016). This has implications for the evaluation of services and care given to older people. How do we assess older peoples’ assessments of their housing quality? Can we be sure that we are measuring residential satisfaction if their assessment of the quality of their accommodation is in fact an assessment of a caring architecture; that is, care and accommodation together?

## Conclusions

This paper concludes that resident-centred care depends on the associations with components in the assemblage in which it is carried out. Architectural space is a strong actor in these processes and negotiations. Architecture does things to people (Gieryn, 2002), although not as a person acting, but in that lived experience is altered (Brott, 2013). This means that neither care nor architecture can be considered alone. It is in their co-emergence as a caring architecture that roles of space and care are constituted and sometimes visible. This is the major generalization that it is possible to infer from this study; an analytic generalization from a multiple-case study, aimed at examining variation or similarities (Polit & Beck, 2010). Thus, on a theoretical level, the study suggests that the different embedded concepts that were examined contribute to different contours of the concept *resident-centred care* (Deleuze & Guattari, 1994). The limitation of this generalization is that there are only two architectural settings in which resident-centred care was examined in this study. In order to fully understand the impact of architectural space, it is necessary to scrutinize each concept—residents’ choice, individualized care and residents’ autonomy—in different contexts. The generalized results should be explored in other architectural conditions in order to expand and deepen this theoretical contribution further (Morse, 1999). Moreover, only three aspects of resident-centred care were in focus in this study. This multidimensional and complex concept does have other features that can be subject to similar research. It is thus suggested, in order to address the limitations of this study, that other dimensions of resident-centred care also be examined in relation to space. The study suggests that each individual concept gets its form and meaning situated in relations in an assemblage of people, spaces and materiality in sometimes highly unstable and volatile situations, processes of exchange, varying mutuality and associations, contributing to a flow of contingent circumstances in which resident-centred care emerges. Resident-centred care is thus always on the move, unpredictable and ambiguous, situated in architectural space.

## References

[CIT0001] Adler P. A., & Adler P. (1994). Observational techniques In Denzin N. K. & Lincoln Y. S. (Eds.), *Handbook of qualitative research* (pp. 377–13). Thousand Oakes: Sage Publications.

[CIT0002] Bromley E. (2012). Building patient-centeredness: Hospital design as an interpretive act. *Social Science & Medicine*, 75(6), 1057–1066. 22703887 10.1016/j.socscimed.2012.04.037

[CIT0003] Brott S. (2013). *Architecture for a free subjectivity: Deleuze and Guattari at the horizon of the real*. Farnham: Ashgate Publishing.

[CIT0004] Brownie S., & Nancarrow S. (2013). Effects of person-centered care on residents and staff in aged-care facilities: A systematic review. *Clinical Interventions in Aging*, 8, 1–10. 23319855 10.2147/CIA.S38589 PMC3540911

[CIT0005] Czarniawska B. (2007). *Shadowing and other techniques for doing fieldwork in modern societies*. Malmö: Liber.

[CIT0006] DeLanda M. (2006). *A new philosophy of society. Assemblage theory and social complexity*. London: Bloomsbury.

[CIT0007] DeLanda M. (2016). *Assemblage theory*. Edinburgh: Edingburgh University Press.

[CIT0008] Deleuze G., & Guattari F. (1994). *What is philosophy?* New York: Colombia University Press.

[CIT0009] Deleuze G., & Guattari F. (2004). *A thousand plateaus: Capitalism and schizophrenia*. London: Continuum.

[CIT0010] Dovey K. (2013). Assembling architecture In Frichot H. & Loo S. (Eds.), *Deleuze and architecture*. Edinburgh: Edinburgh University Press.

[CIT0011] Duff C. (2014). *Assemblages of health. Deleuze’s empiricism and the ethology of life*. Rotterdam: Springer.

[CIT0012] Essén A. (2008). Variability as a source of stability: Studying routines in the elderly home care setting. *Human Relations*, 61(11), 1617–1644.

[CIT0013] Foley R. (2011). Performing health in place: The holy well as a therapeutic assemblage. *Health & Place*, 17(2), 470–479. 21195654 10.1016/j.healthplace.2010.11.014

[CIT0014] Gieryn T. F. (2002). What buildings do. *Theory and Society*, 31(1), 35–74.

[CIT0015] Jacobs J. M. (2006). A geography of big things. *Cultural Geographies*, 13(1), 1–27.

[CIT0016] Kane R. A., Lum T. Y., Cutler L. J., Degenholtz H. B., & Yu T. C. (2007). Resident outcomes in small‐house nursing homes: A longitudinal evaluation of the initial green house program. *Journal of the American Geriatrics Society*, 55(6), 832–839. 17537082 10.1111/j.1532-5415.2007.01169.x

[CIT0017] Lather P., & St. Pierre E. A. (2013). Post-qualitative research. *International Journal of Qualitative Studies in Education*, 26(6), 629–633.

[CIT0018] Law J. (2004). *After method: Mess in social science research*. London: Routledge.

[CIT0019] Li J., & Porock D. (2014). Resident outcomes of person-centered care in long-term care: A narrative review of interventional research. *International Journal of Nursing Studies*, 51(10), 1395–1415. 24815772 10.1016/j.ijnurstu.2014.04.003

[CIT0020] Loue S. (2002). Ethical issues in informed consent in the conduct of research with aging persons In Kapp M. B. (Ed.), *Issues in conducting research with and about older persons* (pp. 3–17). New York: Springer Publishing Company.

[CIT0021] McCormack B., Dewing J., Breslin L., Coyne-Nevin A., Kennedy K., Manning M., … Slater P. (2010). Developing person-centred practice: Nursing outcomes arising from changes to the care environment in residential settings for older people. *International Journal of Older People Nursing*, 5(2), 93–107. 20925711 10.1111/j.1748-3743.2010.00216.x

[CIT0022] McCormack B., & McCance T. V. (2006). Development of a framework for person-centred nursing. *Journal of Advanced Nursing*, 56(5), 472–479. 17078823 10.1111/j.1365-2648.2006.04042.x

[CIT0023] McCormack B., & McCance T. V. (2010). *Person-centred nursing. Theory and practice*. Chichester, West Sussex: Wiley-Blackwell.

[CIT0024] Mol A., Moser I., & Pols J. (Eds.). (2010). *Care in practice. On tinkering in clinics, homes and farms*. Bielefeld: Transcript.

[CIT0025] Molony S. L., Evans L. K., Sangchoon J., Rabig J., & Straka L. A. (2011). Trajectories of at-homeness and health in usual care and small-house nursing homes. *The Gerontologist*, 51(4), 504–515. 21482589 10.1093/geront/gnr022

[CIT0026] Morgan S., & Yoder L. H. (2012). A concept analysis of person-centered care. *Journal of Holistic Nursing*, 30(1), 6–15. 21772048 10.1177/0898010111412189

[CIT0027] Morse J. M. (1999). Qualitative generalizability. *Qualitative Health Research*, 9(1), 5–6.

[CIT0028] Nolan M. R., Davies S., Brown J., Keady J., & Nolan J. (2004). Beyond ‘person‐centred’care: A new vision for gerontological nursing. *Journal of Clinical Nursing*, 13(s1), 45–53. 15028039 10.1111/j.1365-2702.2004.00926.x

[CIT0029] Nord C. (2013). Design according to the law: Juridical dimensions of architecture for assisted living in Sweden. *Journal of Housing and the Built Environment*, 28(1), 147–155.

[CIT0030] Nord C. (2016). Free choice in residential care for older people – a philosophical reflection. *Journal Of Aging Studies*, 37, 59-68. doi:10.1016/j.jaging.2016.02.003 27131279

[CIT0031] Nord C. (2017). Stratum architecture - an interated architectural assemblage of care for the very aged In Nord C. & Högström E. (Eds.), *Caring architecture* (pp. 65–81). Newcastle upon Tyne: Cambridge Scholars Publishing.

[CIT0032] Nord C., & Högström E. (Eds.). (2017). *Caring architecture*. Newcastle Upon Tyne: Cambridge Scholars Publishing.

[CIT0033] O’Toole P., & Were P. (2008). Observing places: Using space and material culture in qualitative research. *Qualitative Research*, 8(5), 616–634.

[CIT0034] Polit D. F., & Beck C. T. (2010). Generalization in quantitative and qualitative research: Myths and strategies. *International Journal of Nursing Studies*, 47(11), 1451–1458. 20598692 10.1016/j.ijnurstu.2010.06.004

[CIT0035] Regnier V. (2002). *Design for assisted living: Guidelines for housing the physically and mentally frail*. New York: John Wiley & Sons.

[CIT0036] SFS (2001:453). *Social services act*. Stockholm: Ministry of Health and Social Affairs.

[CIT0037] Vetenskapsrådet (2002). *Forskningsetiska principer inom humanistisk-samhällsvetenskaplig forskning. [Ethical principles for humanistic and social sicentific research]*. Stockholm: Vetenskapsrådet.

[CIT0038] Yee D. L., Capitman J. A., Leutz W. N., & Sceigaj M. (1999). Resident-centered care in assisted living. *Journal of Aging & Social Policy*, 10(3), 7–26. 10537983 10.1300/J031v10n03_02

